# Inflammation-based scores in a large cohort of adrenocortical carcinoma and adrenocortical adenoma: role of the hormonal secretion pattern

**DOI:** 10.1007/s40618-024-02426-y

**Published:** 2024-07-04

**Authors:** A. Mangone, V. Favero, A. Prete, Y. S. Elhassan, M. Asia, R. Hardy, G. Mantovani, I. Chiodini, C. L. Ronchi

**Affiliations:** 1 Department of Clinical Sciences and Community Health, University of Milan, Milan, Italy; 2 Endocrinology Unit, Fondazione IRCCS Ca’ Granda Ospedale Maggiore Policlinico, Milan, Italy; 3 Department of Biotechnology and Translational Medicine, University of Milan, Milan, Italy; 4 Endocrinology Unit, Ospedale Niguarda Ca’ Granda, Milan, Italy; 5 Institute of Metabolism and Systems Research, University of Birmingham, Edgbaston, B152TT UK; 6 Centre for Endocrinology, Diabetes and Metabolism, Birmingham Health Partners, Birmingham, UK; 7 Department of Endocrinology, Queen Elizabeth Hospital Birmingham, University Hospitals Birmingham NHS Foundation Trust, Birmingham, UK; 8 Institute of Clinical Sciences, University of Birmingham, Birmingham, UK

**Keywords:** Adrenocortical tumors, Inflammation scores, Cushing’s syndrome, Adrenal cancer, Neutrophil–lymphocyte-ratio

## Abstract

**Background:**

Serum inflammation-based scores can predict clinical outcome in several cancer types, including adrenocortical carcinoma (ACC). It is unclear whether the extent of inflammation-based scores alterations in ACC reflects malignancy, steroid excess, or both.

**Methods:**

We investigated a large retrospective cohort of adrenocortical adenomas (ACA, *n* = 429) and ACC (*n* = 61) with available baseline full blood count and hormonal evaluation. We examined the relationship between different inflammation-based scores [neutrophil-to-lymphocyte ratio (NLR), platelet-to-lymphocyte ratio (PLR), lymphocyte-to-monocyte ratio (LMR), systemic immune-inflammation index (SII), and prognostic nutrition index (PNI)] and both malignancy and steroid secretion patterns.

**Results:**

All inflammation-based scores differed between ACC and ACA: patients with ACC had higher NLR, PLR, SII and lower LMR and PNI levels compared to ACA (all *p* values < 0.001). NLR showed a positive correlation with cortisol levels after overnight 1 mg-dexamethasone suppression test (1 mg-DST), both in ACC and ACA (*p* < 0.01). The ROC curve analysis determined NLR > 2.6 as the best cut-off to discriminate ACC from ACA [AUC = 0.846, *p* < 0.01]. At multivariable analysis, NLR > 2.6 was independently associated with ACC, 1 mg-DST cortisol levels and age, but not with tumour size. Considering the ACC, NLR and SII were higher and PNI was lower in patients with cortisol excess compared to those without cortisol excess (*p* = 0.002, *p* = 0.007, and *p* = 0.044 respectively). Finally, LMR and NLR differed between inactive-ACC (*n* = 10) and inactive-ACA (*n* = 215) (*p* = 0.040 and *p* = 0.031, respectively).

**Conclusion:**

Inflammation-based scores are related to steroid secretion both in ACC and ACA. ACCs present a higher grade of inflammation regardless of their hormonal secretion, likely as a feature of malignancy itself.

**Supplementary Information:**

The online version contains supplementary material available at 10.1007/s40618-024-02426-y.

## Introduction

In recent years, serum inflammation-based scores, such as neutrophil-to-lymphocyte-ratio (NLR), platelet-to-lymphocyte-ratio (PLR) and lymphocyte-to-monocyte ratio (LMR), have been extensively studied in oncological patients as they reflect cancer-related systemic inflammation, which is proven to affect the tumour microenvironment [1, 2]. In particular, immune cells contribute to multiple cancer hallmark capabilities by supplying bioactive molecules including cytokines, chemokines, growth factors, reactive oxygen and nitrogen species, and influence every step of the tumorigenesis, from initiation through angiogenesis, invasion and metastasis [3]. Inflammation-based scores have shown a strong independent prognostic value in several cancer types, with the advantage of being easily available in all patients through routine blood examinations [4]–[7]. In adrenocortical carcinoma (ACC), inflammation-based scores have been demonstrated to predict clinical outcomes in patients with both localized disease—i.e. undergoing resection of primary tumour [8]–[11]—and advanced disease treated with systemic pharmacological therapy [12, 13]. Moreover, studies in small patient cohorts evaluating different types of adrenal tumours showed significantly higher inflammation-based scores in ACC than in benign adrenocortical adenomas [14]–[17], highlighting these scores as potential diagnostic markers. However, adrenal tumours represent a very peculiar entity as they can present with autonomous steroid secretion, which can affect the immune response and consequently serum immune scores. Notably, cortisol excess can cause several haematological alterations, such as neutrophilia, lymphopaenia and eosinopaenia, leading to an increased susceptibility to infections, which typically characterizes Cushing’s syndrome (CS) [18]. Indeed, immune cell counts were recently demonstrated to correlate with the degree of hypercortisolism [14], and alterations in inflammation-based scores have been recently found also in patients with mild autonomous cortisol secretion (MACS) [19].

With around 65% of ACCs being hormonally active, and most of them secreting cortisol in excess (either alone or in combination with other steroid hormones, together constituting around 50% of ACCs) [20, 21], it is unclear whether the changes in inflammation-based scores in ACC primarily reflect malignancy, a more aggressive tumour behavior, a generalized presentation of steroid excess, or a combination of both factors.

Therefore, we investigated the complex relationships occurring between inflammation-based scores (used as surrogates of systemic inflammation), malignancy and steroid patterns in adrenocortical tumours.

## Material and methods

We performed a retrospective monocentric study involving a large cohort of 490 patients with adrenocortical tumours referred and followed up in the Adrenal Tumour Service at the Queen Elizabeth Hospital Birmingham (UK) between 2005 and 2022. These included 429 patients with adrenocortical adenoma (ACA) and 61 with ACC. Inclusion criteria were age ≥ 18 years, available full blood count (FBC) at the time of diagnosis and before any active intervention (either adrenalectomy or cortisol-lowering medications or anti-cancer treatments), available baseline hormonal work-up and clinical assessment, and radiological characteristics of the adrenal mass. Patients with phaeochromocytoma and other non-cortical benign or malignant adrenal masses were excluded. Patients with conditions that could affect FBC values at the time of blood testing, such as sepsis and other known infections, haematological diseases, severe cardiomyopathy, active malignancies other than ACC, active autoimmune diseases, or treatment with glucocorticoids or other immunomodulatory drugs, were also excluded. Institutional review board approval for retrospective data review from patients undergoing routine clinical care was obtained from the University Hospital Birmingham NHS Foundation Trust (reference CARMS-18109). Part of the sub-cohort of benign tumours (*n* = 375) was already included in a previous paper from our group focusing on the correlation among inflammation and the degree of cortisol excess in ACA [19], yet with different study design and aims.

All patients underwent clinical assessment at the time of referral which included demographics and past medical history, including the presence of comorbidities potentially related to cortisol excess—such as hypertension and type 2 diabetes (clinical outcomes defined as per [22], and evaluation of CS-related signs and symptoms. Tumours were classified in ACA or ACC based on histology when available and/or radiological and biochemical characteristics according to current guidelines [23, 24]. Tumor size was defined as the maximum tumour diameter.

We recorded hormonal work-up data including morning cortisol following 1-mg overnight dexamethasone-suppression test (1 mg-DST), 24 h urine free cortisol (UFC), late-night salivary cortisol when available, basal plasma adrenocorticotropic hormone (ACTH) when available, aldosterone and plasma renin concentration (only in patients with concomitant hypertension or unexplained hypokalemia), plasma metanephrine and normetanephrine [24], adrenal androgens (when appropriate [23]). Tumours were further classified according to steroid pattern as follows: aldosterone-producing adenomas (APA), cortisol-producing adenomas with Cushing's syndrome (CPA-CS), mild autonomous cortisol secretion (MACS), inactive adenomas (inactive-ACA), inactive-ACC, androgen producing-ACC (androgen-ACC), MACS-ACC, and CS-ACC, according to the most recent European guidelines [24]. CS was defined by the presence of clinical features and more than one positive screening test for hypercortisolism (late-night salivary cortisol, 1 mg-DST, UFC [25, 26]), MACS was defined as the failure to suppress cortisol after 1 mg-DST (cortisol levels above 50 nmol/L) without any evident clinical features of Cushing's syndrome.

The inflammation-based scores were calculated starting from FBC and serum albumin as follows: NLR by dividing the absolute neutrophil count by the absolute lymphocyte count; PLR by dividing the absolute platelet count by the absolute lymphocyte count; SII by multiplying the absolute platelet count and NLR; LMR by dividing the absolute lymphocyte count by the absolute monocyte count; prognostic nutritional index (PNI) by the formula [albumin level (g/L) + (5 × total lymphocyte count)]; NPS giving a score of 0 if neutrophils ≤ 7.5 × 109/L and platelets ≤ 400 × 109/L, a score of 1 if neutrophils > 7.5 × 109/L or platelets > 400 × 109/L, or a score of 2 if neutrophils > 7.5 × 109/L and platelets > 400 × 109/L [27, 28] (Supplementary Table 1).

### Statistical analysis

Continuous data are shown as median and interquartile range (IQR) (25th–75th percentile). Categorical variables are expressed as numbers and percentages. The comparison of non-parametric continuous data was performed by using Mann–Whitney test or Kruskall-Wallis test followed by Dunn’s post-hoc test. The categorical variables were compared by χ2 test or Fisher test, as appropriate.

Spearman’s correlation was performed to test the correlation between cortisol after 1 mg-DST and any inflammation-based scores. For the inflammation-based scores reported as continuous variables that were different in ACC and ACA groups, Quade nonparametric analysis of covariance has been used to adjust for age, tumour maximum diameter and cortisol after 1 mg-DST or UFC. For the inflammation-based scores that were found to be different between ACC and ACA and from inactive-ACC and inactive-ACA, the Receiver Operator Characteristics (ROC) curves were performed to establish the optimal cut-off values and their associated sensitivities, specificities, and areas under the curve (AUC) for distinguishing ACC from ACA and from inactive-ACC from inactive-ACA. The Youden’s index (J = sensitivity + specificity − 1) was used to identify the most appropriate cut-off.

Multivariate logistic regression was used to estimate the odds of having an NLR > 2.6, evaluated with adjustment for potential confounders (presence of ACC, cortisol levels after 1 mg-DST, age, and tumour size).

Two-way ANOVA was performed to assess the interaction between cortisol levels after DST > 50 nmol/L and presence of ACC in influencing NLR values.

P-values of less than 0.05 were considered statistically significant.

Statistical analysis was performed by SPSS version 28.0 statistical package (IBM Corporation), JMP (JMP® Pro, Version 16. SAS Institute Inc., Cary, NC, 1989–2021), and GraphPad Prism version 9 (GraphPad Software).

## Results

### Characteristics of the study cohort

A total of 490 patients was included, divided into 429 patients with ACA (87.55%) and 61 with ACC (12.45%). Among them, 59.5% were women (56.9% in the ACA group and 54.1% in the ACC group). Patients with ACA comprised patients with APA (*n* = 54), CPA-CS (*n* = 22), MACS (*n* = 138), and inactive-ACA (*n* = 215).

The demographic, hormonal, and radiological data are detailed in Table 1. The groups differed, as expected, in terms of age, size, and cortisol secretion; patients with ACC had higher levels of cortisol after 1 mg-DST and higher UFC levels. Five patients with APA had also a cortisol co-secretion, based on cortisol levels after 1mgDST (range 54–90 nmol/L). Since no other hormonal and clinical differences were found between these patients and APA patients without cortisol excess, these subjects were consider as primarily affected by APA and they were included in the APA group.

**Table 1:** Demographic, clinical, laboratory, and radiological data as well as inflammation-based scores in patients with adrenocortical adenomas (ACA) or adrenocortical carcinoma (ACC)

	Total (*n* = 490)	ACA (*n* = 429)	ACC (*n* = 61)	*p* value
Demographics				
Age, years (IQR)	59.5 (49–70)	60 (50–69.5)	54 (41.5–70.5)	**0.04**
Women, *n* (%)	277 (56.5)	244 (56.9)	33 (54.1)	0.68
BMI, kg/m^2^ (IQR)	29.03 (25.1–33.0)	29.1 (25.1–33.1)	29 (25.8–33)	0.79
Comorbidities				
Hypertension, *n* (%)	269 (58.6)	241(60.3)	29 (47.6)	0.06
Unknown, *n*	31			
Diabetes, *n* (%)	102 (22.2)	93 (23.2)	9 (15.3)	0.17
Unknown, *n*	30	28		
Hormonal pattern				
Inactive, *n* (%)	225 (45.9)	215 (50.1)	10 (16.4)	** < 0.001**
APA, *n* (%)	54 (11)	54 (12.6)	NA	**–**
CS, *n* (%)	22 (7.3)	22 (5.1)	14 (22.9)	** < 0.001**
MACS, *n* (%)	138 (33.8)	138 (32.2)	28 (45.9)	**0.02**
androgen-secreting, *n* (%)	9 (1.8)	NA	9 (14.8)	**–**
Cortisol secretion				
Cortisol after 1 mg-DST, nmol/L (IQR)	46 (30–100)	44 (29–81)	340.5 (45.5–703)	** < 0.001**
Unknown, *n*	55	34	21	
24 h—Urinary free cortisol	72 (45–115)	69 (44–108)	193.5 (62–608.3)	** < 0.001**
Unknown, *n*	165	138	27	
Tumour characteristics				
Size, cm (IQR)^§^	2.4 (1.6–3.5)	2.1 (1.5–3)	12.9 (8.5–15.8)	** < 0.001**
Ki67%		NA	20 (6.5–30)	**–**
Unknown, *n*			24	
ENSAT Stage 1/2/3/4		NA	0/22/15/24	**–**
(%)			0/36/24.6/39.4	
Inflammation-based scores				
NLR	2.60 (1.85–3.59)	2.42 (1.74–3.31)	6 (3.15–8.7)	< 0.001*
PLR	143.15 (106.17–186.42)	139.29 (101.74–178.5)	193.2 (137.5–282.74)	< 0.001*
SII	655.69 (450.52–956.09)	606.67 (417.68–877.2)	1463 (810.68–2558.27)	< 0.001*
LMR	3.33 (2.42–4.41)	3.5 (2.6–4.5)	2.0 (1.37–2.75)	< 0.001*
PNI	51.47 (47–55.5)	52 (48.5–56)	45.25 (41.61–51)	< 0.001*
Unknown, *n*	144	139	5	
NPS 0/1/2	423/58/8	302/33/4	31/25/4	** < 0.001**
(%)	(86.5/11.9/1.6)	(91.4/7.7/0.9)	(51.7/41.7/6)	

Categorical variables are reported as *N* (%); statistical comparison was performed by chi-square test. Continuous variables are reported as median (IQR) and statistical analysis were performed by Mann–Whitney test. *p < 0.05 after adjusting for age, tumour maximum diameter and cortisol after 1 mg-DST with Quade nonparametric analysis of covariance

*n* number, *ACA* Adrenocortical adenomas, *ACC* adrenocortical carcinomas, *BMI* body mass index, *1 mg-DST* 1 mg overnight dexamethasone suppression test, *APA* aldosterone-producing adenomas, *CS* Cushing's syndrome, *MACS* mild autonomous cortisol secretion, *NA* not applicable, *NLR* Neutrophil-to-Lymphocyte Ratio, *PLR* Platelet-to-Lymphocyte Ratio, *SII* Systemic Immune-Inflammation Index, *LMR* Lymphocyte-to-Monocyte Ratio, *PNI* Prognostic Nutrition Index, *NPS* Neutrophil-Platelet Score

### Correlations between inflammation-based scores and cortisol secretion levels

**Fig. 1:**
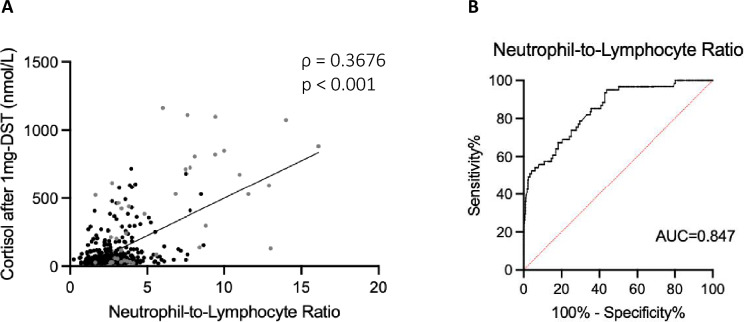
**A** Correlation between cortisol levels after 1-mg overnight dexamethasone-suppression test and neutrophil/lymphocyte ratio (NLR). p-values were determined with Spearman’s correlation coefficient. Gray dots represent patients with adrenocortical carcinoma (ACC, *n* = 61), black dots represent patient with adrenocortical adenoma (ACA, *n* = 429). **B** Area under the curve (AUC) of the neutrophil/lymphocyte ratio (NLR) for distinguishing adrenocortical adenomas (ACA) from adrenocortical carcinomas (ACC)

All inflammation-based scores displayed a significant, although weak, correlation with cortisol levels after 1 mg-DST when considering patients altogether (Table 2). In particular, NLR exhibited the best correlation with cortisol levels after 1 mg-DST, (ρ = 0.3676, *p* < 0.001, as shown in Fig. 1A). This correlation remained significant even when analyzing ACC and ACA patients separately (ρ = 0.6388, *p* < 0.001 for ACC; ρ = 0.2905, *p* < 0.001 for ACA, respectively) (Table 2). UFC showed a positive correlation with NLR (ρ = 0.2675, *p* < 0.001) and SII (ρ = 0.2272, *p* < 0.001), and a negative correlation with LMR (ρ = − 0.1150, *p* = 0.038). The correlation between UFC and NLR (ρ = 0.5553, *p* < 0.001 for ACC; ρ = 0.1811, *p* = 0.002 for ACA, respectively) and SII (ρ = 0.3632, *p* = 0.0348 for ACC; ρ = 0.1391, *p* = 0.002 for ACA, respectively) remained significant when analyzing ACC and ACA patients separately (Table 2).

**Table 2:** Correlations between multiple inflammation-based scores and cortisol levels after 1 mg overnight dexamethasone suppression test

	Total (*n* = 490)		ACA (*n* = 429)		ACC (*n* = 61)	
	ρ Spearman	*p* value	ρ Spearman	*p* value	ρ Spearman	*p* value
NLR	0.3676	** < 0.001**	0.2905	** < 0.001**	0.6388	** < 0.001**
PLR	0.0968	**0.043**	0.0255	0.612	0.2074	0.199
SII	0.3311	** < 0.001**	0.2441	** < 0.001**	− 0.5256	** < 0.001**
LMR	-0.3015	** < 0.001**	− 0.2357	** < 0.001**	0.5212	** < 0.001**
PNI	-0.1694	**0.003**	− 0.0408	0.513	− 0.3633	**0.021**

Correlations were performed using Spearman test

*ACA* adrenocortical adenoma, *ACC* adrenocortical carcinoma, *NLR* Neutrophil-to-Lymphocyte Ratio, *PLR* Platelet-to-Lymphocyte Ratio, *SII* Systemic Immune-Inflammation Index, *LMR* Lymphocyte-to-Monocyte Ratio, *PNI* Prognostic Nutrition Index

### Inflammation-based scores in benign and malignant adrenal tumours

Patients with ACC had higher levels of NLR, PLR, SII and lower levels of LMR and PNI compared to patients with ACA (all *p* values < 0.001), as shown in Table 1. Importantly, this difference remained significant after adjusting age, tumour maximum diameter, and cortisol after 1 mg-DST (Table 1).

Among the inflammation-based scores, NLR demonstrated the highest accuracy in distinguishing ACC from ACA, with an area under the curve (AUC) of 0.847 (95% CI 0.795–0.894) and an optimal cut-off value of 2.6 (Table 3 and Fig. 1B).

**Table 3:** Receiver Operator Curves (ROC) with their most discriminant values (Youden’s Index) for multiple inflammation-based scores to distinguish patients with adrenocortical adenomas (ACA, *n* = 429) from adrenocortical carcinomas (ACC, *n* = 61)

	AUC	*p* value	95% CI		Youden’s Index		
			Lower limit	Upper limit	Value	Sensitivity (%)	Specificity (%)
NLR	**0.847**	** < 0.001**	0.795	0.894	2.6	95.1	56.2
PLR	0.724	** < 0.001**	0.653	0.794	214.2	47.5	88.1
SII	0.837	** < 0.001**	0.781	0.892	1219.7	62.3	91.2
LMR	0.784	** < 0.001**	0.716	0.851	2.71	75.4	72.3
PNI	0.746	** < 0.001**	0.668	0.824	45.5	55.4	88.9

*AUC* area under the curve, *CI* confidence interval, *NLR* Neutrophil-to-Lymphocyte Ratio, *PLR* Platelet-to-Lymphocyte Ratio, *SII* Systemic Immune-Inflammation Index, *LMR* Lymphocyte-to-Monocyte Ratio, *PNI* Prognostic Nutrition Index

**Fig. 2:**
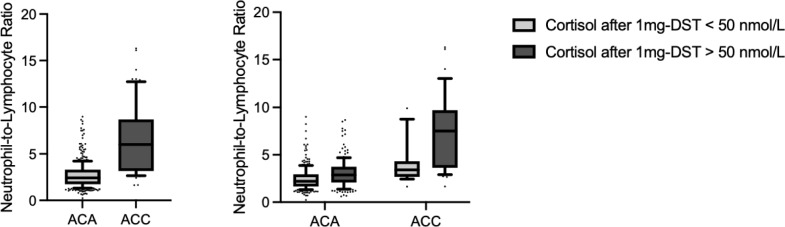
Neutrophil/lymphocyte ratio (NLR) in patients with adrenocortical adenomas (ACA) and adrenocortical carcinomas (ACC). 1-mg DST = 1-mg overnight dexamethasone-suppression test. Data are shown as median and interquartile range, the upper and the lower whiskers represent respectively the 90 and the 10 percentiles

The logistic regression analysis showed that the presence of NLR > 2.6 was independently associated with the presence of ACC [OR 7.955 (95% CI 1.374–46.058), p = 0.021], cortisol levels after 1 mg-DST (50 nmol/L increase) [OR 1.336 (95% CI 1.185–1.507), *p* < 0001] and age (1-year increase) [OR 1.035 (95% CI 1.018–1.051, *p* < 0.001] but not with size (1 cm increase) [OR 0.995 (95% CI 1.185–1.507), *p* = 0.950]. The results did not change even including UFC levels in the place of cortisol levels after 1 mg-DST or including the presence of hypertension and diabetes in the model (data not shown). Finally, the two-way ANOVA analysis showed that both the presence of malignancy (ACC *vs* ACA, *p* < 0.001) and the presence of cortisol excess (cortisol after 1 mg-DST > 50 nmol/L, *p* < 0.001) were associated with higher NLR values (Fig. 2). Moreover, the same analysis showed that no significant interaction was present between malignancy and cortisol excess (*p* = 0.21) in influencing NLR values.

### Effect of cortisol excess on inflammation-based scores in adrenocortical carcinomas

The ACC cohort was divided in two groups according to the presence of normal (ACC without cortisol excess, comprising patients with inactive-ACC and androgen-ACC) or pathological levels of cortisol after 1 mg-DST (ACC with cortisol excess, combining both patients with MACS-ACC and CS-ACC), as detailed in Supplementary Table 2.

There were no significant differences in terms of clinical and demographic characteristics between patients with or without cortisol excess, except for ENSAT stage (Table 4). NLR and SII was found to be higher in patients with cortisol excess compared to those without (*p* = 0.002 and *p* = 0.007, respectively). On the other hand, PNI was lower in the subgroup with cortisol excess compared to the one without hypercortisolism (*p* = 0.044) (Table 4).

**Table 4:** Demographic, clinical, laboratory, and radiological data of patients with adrenocortical carcinoma (ACC), divided according to the presence of normal or pathological levels of cortisol after 1 mg-DST

	ACC without cortisol excess (*n* = 19)	ACC with cortisol excess (*n* = 42)	*p* value
Demographics			
Age, years (IQR)	53.5 (47.2–73.5)	55 (38–64.5)	0.554
Women, *n* (%)	8 (42.1)	25 (59.5)	0.122
BMI, kg/m^2^ (IQR)	28.25 (21.75–38.85)	29 (26–33.75)	0.313
Comorbidities			
Hypertension, *n* (%)	7 (38.9)	21 (50)	0.381
Unknown, *n*		0	
Diabetes, *n* (%)	2 (10.5)	7 (17.5)	0.474
Unknown, *n*		2	
Tumour characteristics			
Size, cm (IQR)	12.4 (7.4–13.9)	13 (9.1–16)	0.368
Ki67%	12 (5–22.5)	25.5 (7.75–39.25)	0.067
Unknown, *n*	2	22	
ENSAT Stage 1/2/3/4	0/11/5/3	0/11/10/21	**0.018**
(%)	0/57.9/26.3/15.8	0/26.2/23.8/50	
Inflammation based scores			
NLR	3.4 (2.67–4.32)	7.5 (3.62–9.71)	**0.002**
PLR	178.43 (134.5–216.7)	227.9 (137.5–312.11)	0.194
SII	870.85 (659.36–1516.5)	1796.7 (1076.5–2645.01)	**0.007**
LMR	2.45 (1.65–3.63)	1.67 (1.19–2.63)	0.061
PNI	48 (44.25–55.5)	44 (40.5–50.5)	**0.044**
NPS 0/1/2	13/6/0	19/19/4	0.074
(%)	(68.4%/31.6%/0%)	(45.2%/45.2%/9.6%)	

Categorical variables are reported as N (%), statistical comparison performed by chi-square test. Continuous variables are reported as median (IQR) and statistical analysis were performed by Wilcoxon test

*NLR* Neutrophil-to-Lymphocyte Ratio, *PLR* Platelet-to-Lymphocyte Ratio, *SII* Systemic Immune-Inflammation Index, *LMR* Lymphocyte-to-Monocyte Ratio, *PNI* Prognostic Nutrition Index, *NPS* Neutrophil-Platelet Score

### Assessing inflammation-based scores for distinguishing inactive adrenocortical carcinomas from adenomas

**Fig. 3:**
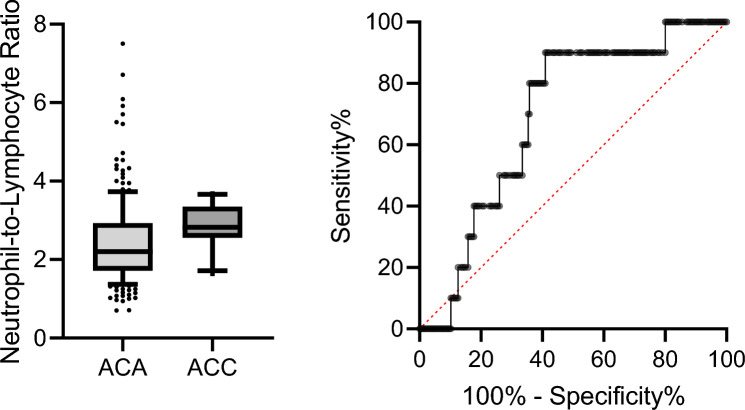
Neutrophil-to-lymphocyte ratio (NLR, **A** and lymphocyte-to-monocyte ratio (LMR, **B** for distinguishing inactive adrenocortical adenomas (ACA) from inactive adrenocortical carcinomas (ACC)

According to our findings, we hypothesized that inflammation-based score might be useful to differentiate inactive ACC from ACA. When specifically analyzing this subgroup of patients, i.e., 215 patients with non-functioning ACA (inactive-ACA) and 10 patients with inactive-ACC, only LMR and NLR were different between the two groups (NLR 2.2 (1.71–2.93) for ACA and 2.82 (2.55–3.35) for ACC, *p* = 0.040, and LMR 3.66 (2.98–4.6) for ACA and for ACC 2.69 (2.31–3.81), *p* = 0.031, respectively). We therefore evaluated the discriminatory power of these two inflammation-based scores in differentiating patients with inactive-ACC and inactive-ACA. Here NLR showed an AUC of 0.692 (95% CI 0.563–0.821); the cut-off value with the best compromise between sensitivity and specificity being 2.42 (Se 90%, Sp 59.1%), while LMR showed an AUC of 0.702 (95% CI 0.540–0.865); the cut-off value with the best compromise between sensitivity and specificity was set at 2.71 (Se 60%, Sp 83%) (Fig. 3).

## Discussion

Our study aimed to investigate multiple inflammation-based scores—as surrogates of systemic inflammation—in a large cohort of patients with different types of adrenocortical tumors and their relationship with the presence of malignancy and cortisol excess. To our knowledge, this is the first study to suggest an independent relationship among adrenocortical malignancy, autonomous cortisol secretion, and degree of systemic inflammation.

Cortisol excess in Cushing's syndrome can have a remarkable impact on immune cells and systemic inflammation. Previous studies reported changes in blood cell counts in patients with endogenous Cushing's syndrome, including an increase in white blood cells and neutrophils and a decrease in lymphocytes; these changes correlated with the severity of the disease and improved after Cushing's syndrome was resolved [29]–[32]. Specifically, chronic hypercortisolism induces lymphopaenia mainly in the CD4^+^ subset, causing an altered CD4/CD8 ratio which increases patients’ susceptibility to infections, and also alters the ratio of Th1/Th2 subpopulations leading to apoptosis of mature T lymphocytes. At the same time, by increasing the neutrophil count, CS contributes to the establishment of a hypercoagulative and chronic inflammatory state, characterized by persistently increased levels of interleukin-1 (IL-1), interleukin-6 (IL-6), and tumor necrosis factor alpha (TNFα) [18]. In our study on patients with adrenocortical tumours with or without cortisol excess, we observed that all evaluated inflammation-based scores correlated, although weakly in some cases, with cortisol levels after 1 mg-DST, overall confirming cortisol excess to be associated with a pro-inflammatory state.

Moreover, oncological patients usually experience a change in peripheral blood cell composition characterized by an expansion of the myeloid components and a reduction of the lymphoid components [33]. This is reflected by modifications of some markers of inflammation, such as the increase of NLR, PLR and SII and the decrease of LMR and PNI. These alterations are considered to be driven by changing circulating cytokines and chemokines profiles released by malignant cells such as TNFα, EGFR ligands, transforming growth factor‐beta (TGF‐β) and IL-6, contributing to local immune evasion and tumor progression [34]. When comparing patients with ACC to patients with ACA, we found significant differences in all inflammation scores between the two groups. In ACC patients, we observed higher values of NLR, PLR, and SII, and lower levels of LMR and PNI, indicating a greater degree of inflammation. The presence of a more remarkable degree of inflammation in ACC patients compared to ACA has been previously described in small studies with unselected populations. In particular, Mochizuki et al. found higher NLR values in patients with malignant adrenal tumors (13 patients, 9 of them with ACC, 4 with lymphoma) compared to a heterogeneous group of patients with benign adrenal tumors (46 patients) [16]. Sisman et al. found similar results in a study in which different inflammation-based scores were evaluated in 13 patients with ACC and 30 patients with benign nonfunctioning tumors [17].

According to our study findings, patients with ACC consistently exhibited a significantly higher level of inflammation regardless of the degree of cortisol secretion. These results align with recent research by Mangion et al., showing that individuals with malignant Cushing's syndrome (including ACC and ectopic Cushing's syndrome) exhibited elevated NLR and SII values and lower LMR values compared to those with benign Cushing's syndrome (arising from pituitary or adrenal adenomas) [15].

Notably, in the present study NLR emerged as the most accurate marker for distinguishing between ACA and ACC, with an AUC of 0.847 and an optimal cut-off value of 2.6. It is worth noting that the same threshold of 2.6 was previously reported by Detomas et al. in a study comparing patients with ACC to those with ACA and mild autonomous cortisol secretion [14]. In our investigation, we did a further step showing that the NLR cut-off value set at 2.6 remained independently associated with both 1-mg DST cortisol levels and malignancy even after adjusting for tumor size and patient age.

To comprehensively assess the independent influence of cortisol secretion, we conducted a subanalysis within the ACC patient group, categorizing them into two distinct subsets: those with and those without cortisol excess. In this subanalysis, NLR and SII levels were higher while LMR levels were lower among patients with cortisol excess. These observations may suggest that the extent of cortisol secretion contributes to exacerbate inflammation among patients with malignancies.

Furthermore, we examined inflammation-based scores separately in patients with hormonally inactive tumors to reaffirm that malignancy itself has an impact on inflammatory status. Notably, our findings highlight that NLR and LMR effectively differentiate between inactive ACC and inactive ACA, indicating their potential use as a diagnostic tool for distinguishing between—sometimes challenging—benign and malignant hormonally inactive tumors.

The present study has some limitations due to its retrospective nature, which prevents us from drawing conclusions on causal effects. Furthermore, data on other circulating inflammation markers, such as C-reactive protein and pro-inflammatory cytokines, were either missing or only available for a small subset of patients. Nevertheless, the main strength of our study lies in the substantial number of patients analyzed and the homogeneity in the measurement of the parameter of interest, both in terms of full blood count and systematic hormonal work-up. However, in our ACC cohort most patients (68.8%) presented cortisol-secreting tumors, either alone or in combination with other hormones, whereas we had only a small number of patients with inactive ACC. The percentage of cortisol-secreting ACC in our sample is slightly higher as compared to that reported in the literature (i.e. 50%) [21, 21]. This discrepancy could be partially due to the fact that we considered cortisol-secreting also cases with biochemical evidence of autonomous cortisol without clinical phenotype of overt Cushing syndrome. Another study limitation is that the subgroups of patients with or without cortisol hypersecretion were unbalanced in terms of ENSAT stage, which could have influenced part of the results. Overall, we recognize that the sub-analysis investigating non-secreting ACC should be considered as preliminary and our findings will need to be confirmed in larger multicentric series. Finally, inflammation-based score data after surgical removal of the adrenal mass, which could have been even more informative, were not available.

Notwithstanding these limitations, our study provides novel insights into the relationship between inflammation, malignancy and endogenous cortisol excess in patients with adrenocortical tumours. We observed that cortisol excess is associated with a pro-inflammatory state, as evidenced by the multiple inflammation-based scores. We also demonstrated that patients with ACC have higher levels of inflammation compared to patients with ACA, with NLR and LMR being the most accurate in distinguishing between inactive ACA and ACC, suggesting their potential as additional differential diagnostic tool for incidentalomas. Further research is required to confirm these findings and explore the clinical implications of inflammation-based scores in managing adrenocortical tumors.

## Supplementary Information

Below is the link to the electronic supplementary material.

Supplementary file1 (DOCX 25 KB)

## Data Availability

The authors confirm that the majority of data supporting the findings of this study are available within the article and/or its supplementary materials. Some data sets generated during and/or analyzed during the current study are not publicly available but are available from the corresponding author on reasonable request.
