# Supplementary material for: Inflammation-based scores in a large cohort of adrenocortical carcinoma and adrenocortical adenoma: role of the hormonal secretion pattern
Source: J Endocrinol Invest. 2024 Jul 4;48(1):81–90. doi: 10.1007/s40618-024-02426-y (PMC11729201; doi:10.1007/s40618-024-02426-y)
Supplement: Supplementary file 1 — Supplementary file1 (DOCX 25 KB) [file 40618_2024_2426_MOESM1_ESM.docx]

**Supplementary Table 1 – Serum inflammation-based scores evaluated in this study**

| **Serum inflammation-based score** | **Abbreviation** | **Formula** |
| --- | --- | --- |
| Neutrophil-to-Lymphocyte Ratio | NLR | Neutrophil count / Lymphocyte count |
| Platelet-to-Lymphocyte Ratio | PLR | Platelet count / Lymphocyte count |
| Systemic Immune-Inflammation Index | SII | Platelet count x Neutrophil count / Lymphocyte count |
| Lymphocyte-to-Monocyte Ratio | LMR | Lymphocyte count / Monocytes count |
| Prognostic Nutrition Index | PNI | albumin level (g/L) + 5 x Lymphocyte count (10^9^/L) |
| Neutrophil-Platelet Score | NPS | 0 if platelet are ≤ 400x10^9^/L AND neutrophils are ≤ 7.5x10^9^/L  1 if platelet are > 400x10^9^/L OR neutrophils are > 7.5x10^9^/L  2 if platelet are > 400x10^9^/L AND neutrophils are > 7.5x10^9^/L |

**Supplementary Table 2 – Demographic, clinical, laboratory, and radiological data of patients with adrenocortical adrenocortical carcinoma (ACC) divided in four groups according to the secretion pattern.**

|  | inactive-ACC  (n=10) | androgens-ACC  (n=9) | MACS-ACC  (n=28) | Cushing-ACC  (n=14) | p value |
| --- | --- | --- | --- | --- | --- |
| **Demographics** |  |  |  |  |  |
| Age, years (IQR) | 64.5 (47.7-79.5) | 52 (41.5-73) | 57 (48-68.5) | 43.5 (33.2-59) | 0.198 |
| Women, n (%) | 4 (40) | 3 (33.3) | 15 (53.6) | 11 (78.6) | 0.122 |
| BMI, kg/m^2^ (IQR) | 28 (27-29.9) | 29 (20.25-39.1) | 30 (25.4 – 34) | 29 (25.9-33.5) | 0.894 |
| **Comorbidities** |  |  |  |  |  |
| Hypertension, n (%)  Unknown, n | 4 (44.4)  1 | 4 (44.4) | 13 (48.2)  1 | 7 (50) | 0.991 |
| Diabetes, n (%)  Unknown, n | 1 (10) | 1 (11.1) | 5 (18.5)  1 | 2 (15.4)  1 | 0.906 |
| **Tumour characteristics** |  |  |  |  |  |
| Size, cm (IQR) | 7.6 85.3-13-6) | 13.4 (9.75-15) | 13 (10-17.9) | 11.5 (8.9-15.2) | 0.221 |
| Ki67%  Unknown, n | 17 (6-32.5)  1 | 8 (4.5-18)  1 | 27 (5.1-60)  15 | 20 (13-37)  7 | 0.204 |
| ENSAT Stage 1/2/3/4  (%) | 0/7/1/2  0/70/10/20 | 0/4/4/1  0/44.4/44.4/11.2 | 0/9/8/11  0/32.1/28.6/39.3 | 0/2/2/10  0/14.3/14.3/71.4 | **0.021** |

Categorical variables are reported as N (%); statistical comparison was performed by chi-square test or Fisher test, as appropriate. Continuous variables are reported as median (IQR) and statistical analysis were performed by Kruskall-Wallis test followed by Dunn’s post-hoc test.

Legend: ACC, adrenocortical carcinomas; BMI, body mass index.
